# Supplementary material for: Deep learning enhanced quantum holography with undetected photons
Source: Photonix. 2024 Dec 18;5(1):40. doi: 10.1186/s43074-024-00155-2 (PMC11655614; doi:10.1186/s43074-024-00155-2)
Supplement: Supplementary file 1 — Supplementary Material 1. Please see Supplementary material for supporting contents including Table 1 and Figs. S1-S10. [file 43074_2024_155_MOESM1_ESM.pdf]

Supplementary Material: Deep Learning Enhanced Quantum Holography with Undetected Photons

Weiru Fan[1†], Gewei Qian[1†], Yutong Wang[2], Chen-Ran Xu[1*], Ziyang Chen[3], Xun Liu[4], Wei Li[4], Xu Liu[5], Feng Liu[2], Xingqi Xu[1*], Da-Wei Wang[1,5,6], Vladislav V. Yakovlev[7*]

[1]Zhejiang Province Key Laboratory of Quantum Technology and Device, School of Physics, and State Key Laboratory for Extreme Photonics and Instrumentation, Zhejiang University, Hangzhou, 310027, Zhejiang Province, China.
[2]College of Information Science and Electronic Engineering, Zhejiang University, Hangzhou, 310027, Zhejiang Province, China.
[3]College of Information Science and Engineering, Fujian Key Laboratory of Light Propagation and Transformation, Huaqiao University, Xiamen, 361021, Fujian Province, China.
[4]Beijing Institute of Space and Electricity, China Academy of Space Technology, Beijing, 100094, China.
[5]College of Optical Science and Engineering, Zhejiang University, Hangzhou, 310027, Zhejiang Province, China.
[6]Hefei National Laboratory, Hefei, 230088, Anhui province, China.
[7]Department of Biomedical Engineering, Texas A&M University, College Station, 77843, TX, USA.

*Corresponding author(s). E-mail(s): crxu@zju.edu.cn; xuxingqi@zju.edu.cn; yakovlev@tamu.edu;
[†]These authors contributed equally to this work.

Table 1 Comparison between different holography modalities

	Single shot	Model free	Reference free	Joint-measurement free	Background resilience	Wavelength conversion
**Classical way**						
TIE [1]	✓		✓	✓		
Off-axis [2]	✓			✓		
Phase shift [3]		✓		✓		
Deep learning [4]	✓	✓	✓	✓		
**Quantum way**						
Polarization entanglement[5]					✓	
QHUP with phase shift [6]		✓		✓	✓	✓
QHUP with off-axis [7]	✓			✓	✓	✓
DL-QHUP (Ours)	✓	✓	✓	✓	✓	✓

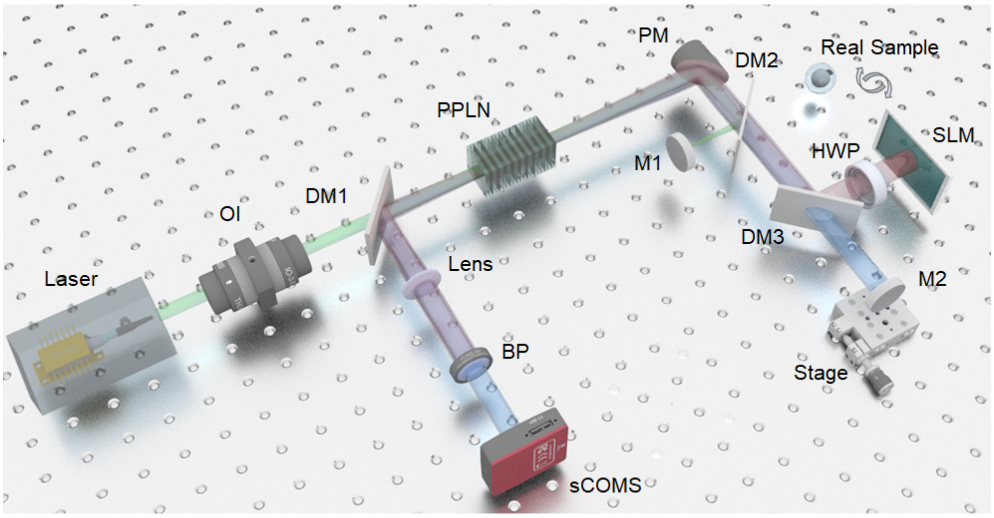

Fig. S1 Experimental setup. The nonlinear crystal (periodically poled lithium niobate; PPLN) is pumped by continuous wave (green) emitted by the laser to produce entangled signal (blue) and idler (red) beams. Afterwards, the pump beam is separated by the dichroic mirror (DM2), while the signal and the idler beams are separated by DM3. The spatial light modulator (SLM) or objects are placed into idler path to acquire training dataset and holograms with real samples. The bandpass filter (BP) is used to select signal photons with certain wavelength, and filter out the idler photons. The off-axis parabolic mirror and lenses are used to collimate and collect lights, and the SLM or objects cannot be imaged on scientific complementary metal-oxide semiconductor (sCMOS) camera. M1 and M2 are mirrors; HWP is half-wave plate; OI is optical isolator.

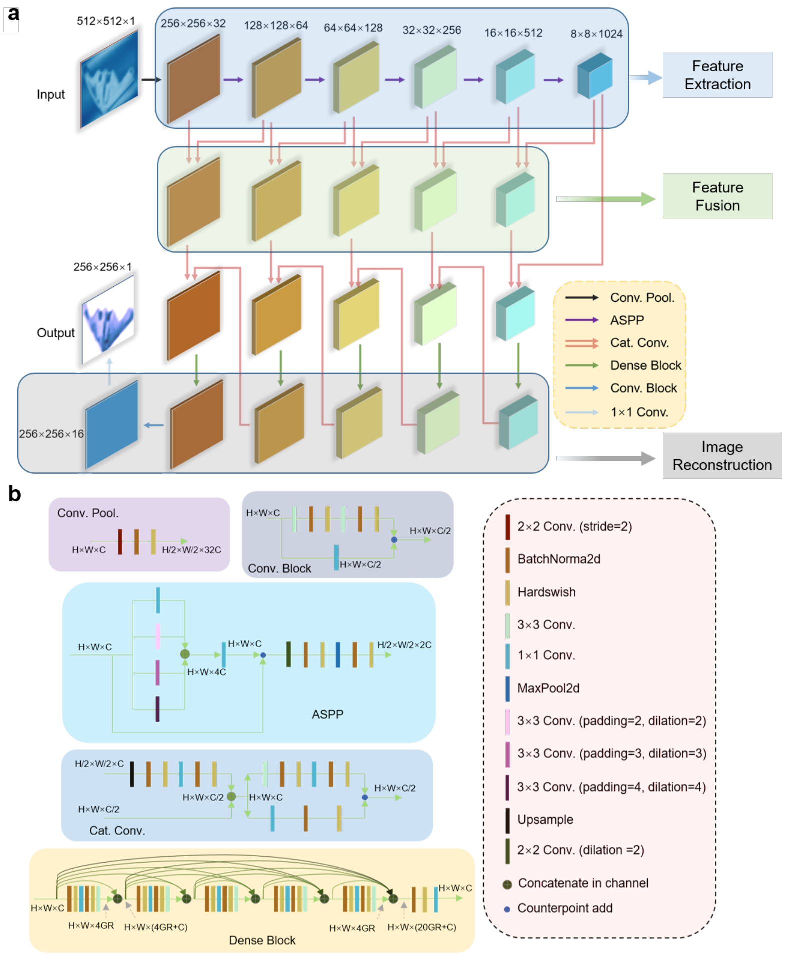

Fig. S2 (a) The architecture and workflow of QHUPnet. The one-shot hologram is fed into QHUPnet as sole input while the output can be adjusted by final 1×1 convolution layer according to expectant image dimension. The block/layer with the same color and volume indicates the same size of the feature map. (b) The details for each module in QHUPnet. n $\times$ n Conv. represents the convolution operation with n $\times$ n kernel. GR is the growth rate and equal to (32, 16, 16, 16, 16) in order. H, W and C are the height, width and channel of the image or feature map, respectively.

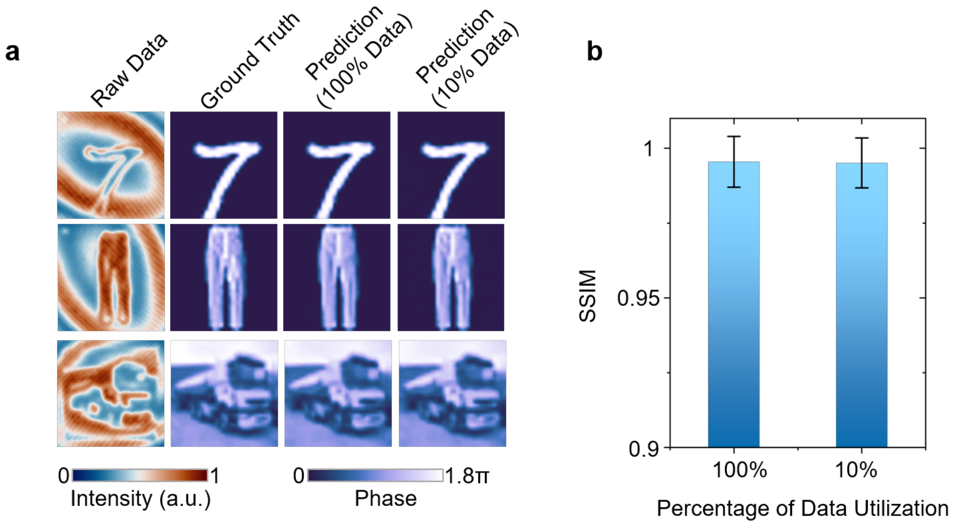

Fig. S3 The performance test of QHUPnet with different sizes of training datasets. QHUPnet holds excellent performance until the the number of training data is reduced to 10%. The size of complete dataset is 150,000 (90% is used to train QHUPnet). Error bar: ± standard deviation.

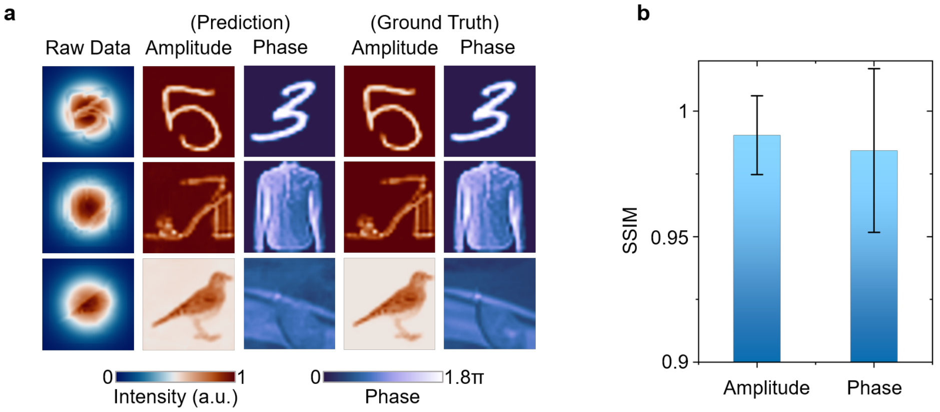

Fig. S4 Imaging complex-amplitude objects by DL-QHUP. (a) Randomly selected ground truth, the corresponding holograms and the predictions of QHUPnet. (b) Performance evaluation of testing dataset by SSIM. Error bar: ± standard deviation. The number of images is 15000.

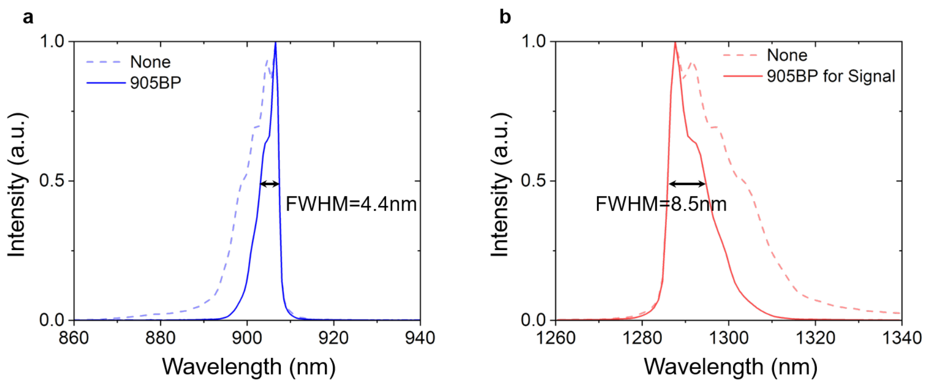

Fig. S5 (a) and (b) are spectra of signal and idler photons, respectively. The blue and red dashed lines denote the original spectra while the blue and red solid lines represent the narrowed spectra of signal and idler photons with a 905 nm bandpass filter inserted in signal path. The full width at half maximum (FWHM) of signal photons is twice the one of idler photons with the bandpass filter.

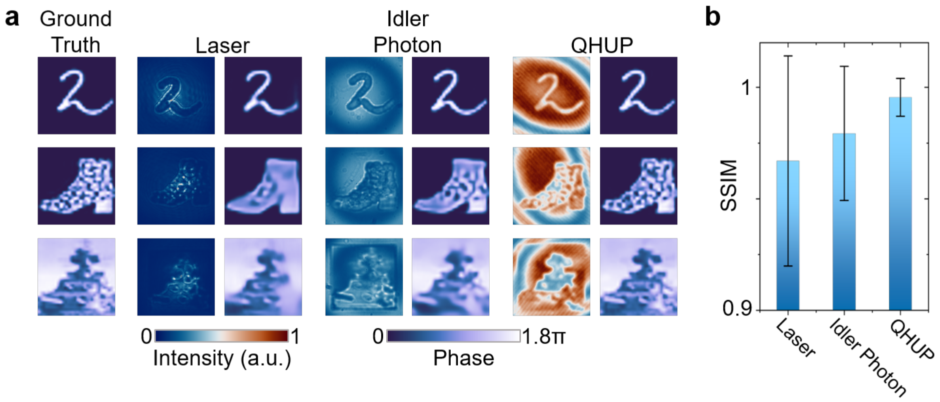

Fig. S6 Imaging performance of different interferometers. (a) Randomly selected ground truth, the corresponding holograms and the predictions of QHUPnet for interferometers with laser, idler photons and QHUP, respectively. (b) Performance evaluation of the testing dataset by SSIM. The predicted results from QHUP outperform other two interferometers. The data obtained by laser interferometer exhibit large variations, resulting from the significant non-local diffraction and crosstalk among pixels of images. Error bar: ± standard deviation. The number of images is 15000.

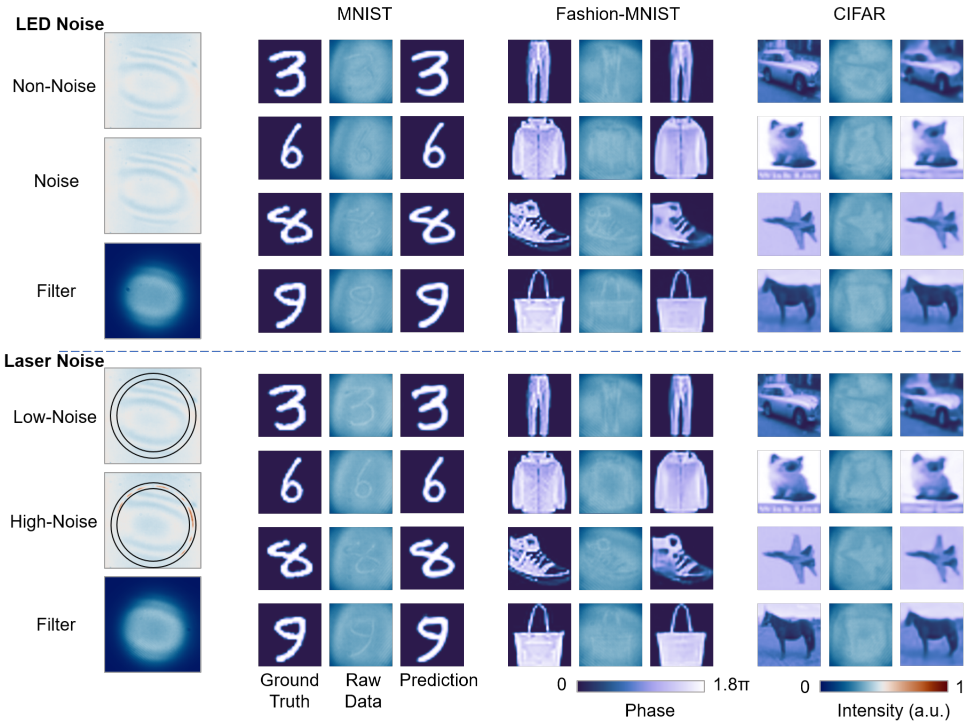

Fig. S7 DL-QHUP performance with background noise. The first column is the raw data with and without the 905 nm bandpass filter for signal photons. The solid ring is the affected area arising from stimulated emission. We set the power of laser in "low-noise" case equal to the power of idler photons, while adjust the powers of laser and LED in the cases of "high-noise" and "LED noise" three orders larger, respectively.

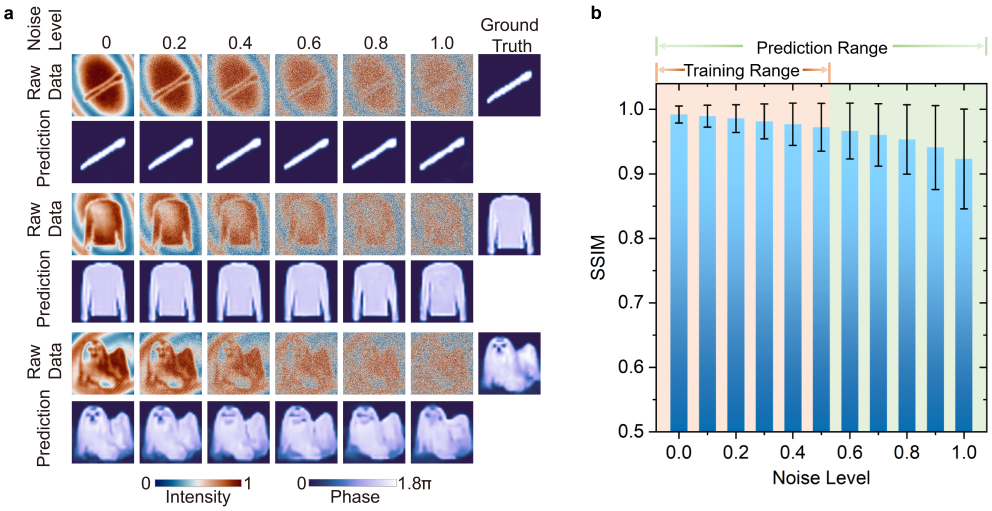

Fig. S8 Evaluation of DL-QHUP under different noise levels. (a) Randomly selected ground truth, the corresponding holograms degraded by random Gaussian noise with different noise levels (variances) and the predictions of QHUPnet. (b) The summary of quantitative results showing performance evaluation using structural similarity index measurement (SSIM) as a function of noise levels. The training stage only covers the noise level below 0.5 (orange region), while prediction contains noise level from 0 to 1 (green region). All simulated holograms are generated by superimposing a random noise sampling from the Gaussian distribution with different levels onto the raw data with noise level = 0. Error bar: ± standard deviation. The number of images is 1500.

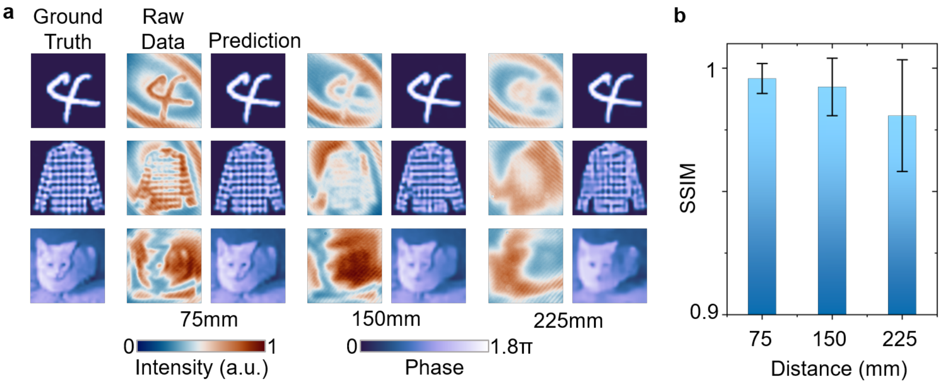

Fig. S9 DL-QHUP imaging at different detection distances. (a) Randomly selected ground truth, the corresponding holograms and the predictions of QHUPnet. (b) Performance evaluation of the testing dataset by SSIM. Although the detection distances deviate from the imaging plane, DL-QHUP can work effectively. For comparison, the data with different detection distances are trained in the same initial weights and epoch. The imaging quality can be improved by increasing the training steps. Error bar: ± standard deviation. The number of images is 15000.

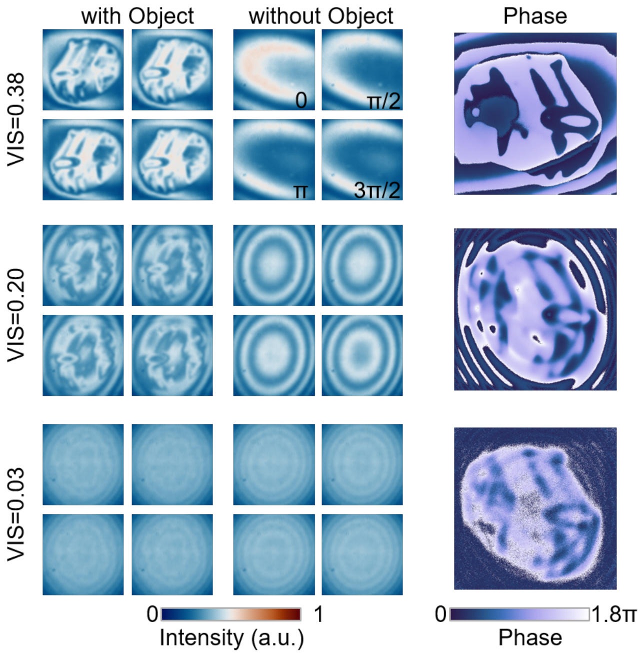

Fig. S10 A set of holograms for the phase retrieval by four-step phase-shifted method with different visibility. The holograms with the object (the first and second columns), the reference holograms without the object (the third and fourth columns) and the final results of phase imaging (the fifth column) are drawn, respectively.

References

[1] Zuo, Chao and Li, Jiaji and Sun, Jiasong and Fan, Yao and Zhang, Jialin and Lu, Linpeng and Zhang, Runnan and Wang, Bowen and Huang, Lei and Chen, Qian. Transport of intensity equation: a tutorial. Opt Laser Eng. 2020; 135, 106187.

[2] Cuche, Etienne and Marquet, Pierre and Depeursinge, Christian. Spatial filtering for zero-order and twin-image elimination in digital off-axis holography. Appl Opt. 2000; 39, 4070-4075.

[3] Schnars, Ulf and Falldorf, Claas and Watson, John and Jüptner, Werner. Digital holography. Berlin: Springer Berlin Heidlerberg 2015.

[4] Rivenson, Yair and Zhang, Yibo and Günaydın, Harun and Teng, Da and Ozcan, Aydogan. Phase recovery and holographic image reconstruction using deep learning in neural networks. Light Sci Appl. 2018; 7, 17141.

[5] Defienne, Hugo and Ndagano, Bienvenu and Lyons, Ashley and Faccio, Daniele. Polarization entanglement-enabled quantum holography. Nat Phys. 2021; 17, 591-597.

[6] Töpfer, Sebastian and Gilaberte Basset, Marta and Fuenzalida, Jorge and Steinlechner, Fabian and Torres, Juan P and Gräfe, Markus. Quantum holography with undetected light. Sci Adv. 2022; 8, eabl4301.

[7] León-Torres, Josué R and Krajinić, Filip and Kumar, Mohit and Gilaberte Basset, Marta and Setzpfandt, Frank and Gili, Valerio Flavio and Jelenković, Branislav and Gräfe, Markus. Off-axis holographic imaging with undetected light. Opt Express. 2024; 32, 35449–35461.
